# Case of Acute Laryngitis

**Published:** 1852-08

**Authors:** 


*Case of Acute Laryngitis.* By Dixi Crosby, M. D., Prof. of Surgery and Obstetrics in Dartmouth College.

There are two distinct forms of this disease recognized by pathologists, viz., mucous and submucous. A case of the mucous variety having occurred recently in this vicinity, possessing more than common interest on account of its progress before medical aid was called, and the unexpected recovery of the patient, I send it for publication in your Journal.

Mr. A. D., 30, was out in the rain, Dec. 31, 1851, from which resulted a "common cold and sore throat." These symptoms increased up to the time I was called, and found that another physician had just bled him some eight or ten ounces, which produced incomplete syncope. He was sitting erect with respiration so loud as to be heard in an adjoining room. Could scarcely articulate in a whisper loud enough to be heard. Had not swallowed for several hours, and refused to try. Confusion of ideas with dizziness, approaching stupor, and perspiration starting from every pore. Cannot protrude the tongue much beyond the teeth.

4, P. M. Four vigorous leeches were applied over the larynx. After they fell off, the throat was enveloped with hot wet cloths to promote the flow of blood. I made a solution of forty grs. nit. silver to the ounce of water, and against every disadvantage, had the good fortune to pass a sponge saturated with the solution into the larynx. Directed more leeches and promote the bleeding as before.

9, P. M. Breathes a little easier. Leech bites, bleeding freely. Pulse quick and feeble. Respiration audible in any part of the room. Passed the sponge as before. Pediluvium.

2d. 8, A. M. Rested but little. Breathes easier. Speaks in a whisper, and asks to have the sponge used again, which I did. Does not swallow any thing. Apply leeches, followed as before, by warm fomentatives, to be followed by cold after leech bites have ceased to bleed.

2, P. M. A little improved. Sleeps semi-recumbent.

9, P. M. General improvement. Has not swallowed, and refuses to try. Says the cold affected him unpleasantly, increasing the difficulty of breathing. Apply a blister on the larynx.

3d. 9, A. M. General improvement; has slept somewhat. Has a good blister. Thinks he will try to swallow a dose of castor oil. Says the sponge relieves him more than every thing else, and hopes I shall pass it again.

10, P. M. Improved in every particular. Oil operated well. Can lie horizontal. Swallows with difficulty; breathes without noise; voice husky.

℞ G. Arabic, ℥ss.
Nit. Potass., ʒii.
Ant. Tart., gr. iii.
Hot water, half a pint.
Dissolve and give a table spoonful every two hours.

4th. Has had a good night. Can swallow with increasing ease, and now for the first time thinks he will take some thin gruel, not having swallowed any food or drank since my first visit. From this time he convalesced rapidly, and is now, Jan. 20th, quite well.

The sponge used was fastened to a crooked whalebone handle.—*New Hampshire Journal of Medicine.*

